# Body composition at birth and its relationship with neonatal anthropometric ratios: the newborn body composition study of the INTERGROWTH-21^st^ project

**DOI:** 10.1038/pr.2017.52

**Published:** 2017-05-31

**Authors:** José Villar, Fabien A Puglia, Tanis R Fenton, Leila Cheikh Ismail, Eleonora Staines-Urias, Francesca Giuliani, Eric O Ohuma, Cesar G Victora, Peter Sullivan, Fernando C Barros, Ann Lambert, Aris T Papageorghiou, Roseline Ochieng, Yasmin A Jaffer, Douglas G Altman, Alison J Noble, Michael G Gravett, Manorama Purwar, Ruyan Pang, Ricardo Uauy, Stephen H Kennedy, Zulfiqar A Bhutta

**Affiliations:** 1 Nuffield Department of Obstetrics and Gynaecology and Oxford Maternal and Perinatal Health Institute, Green Templeton College, University of Oxford, Oxford, UK; 2 Department of Community Health Sciences, Alberta Children’s Hospital Research Institute, University of Calgary, Calgary, Alberta, Canada; 3 Dipartimento di Scienze Pediatriche e dell’Adolescenza, Cattedra di Neonatologia, Università degli Studi di Torino, Torino, Italy; 4 Programa de Pós-Graduação em Epidemiologia, Universidade Federal de Pelotas, Pelotas, Brazil; 5 Department of Paediatrics, University of Oxford, Oxford, UK; 6 Programa de Pós-Graduação em Saúde e Comportamento, Universidade Católica de Pelotas, Pelotas, Brazil; 7 Faculty of Health Sciences, Aga Khan University, Nairobi, Kenya; 8 Department of Family and Community Health, Ministry of Health, Muscat, Sultanate of Oman; 9 Centre for Statistics in Medicine, Nuffield Department of Orthopaedics, Rheumatology and Musculoskeletal Sciences, University of Oxford, Oxford, UK; 10 Department of Engineering Science, University of Oxford, Oxford, UK; 11 Global Alliance to Prevent Prematurity and Stillbirth, Seattle, Washington; 12 Nagpur INTERGROWTH-21^st^ Research Centre, Ketkar Hospital, Nagpur, India; 13 School of Public Health, Peking University, Beijing, China; 14 Division of Paediatrics, School of Medicine, Pontifical Universidad Católica de Chile, Santiago, Chile; 15 Center of Excellence in Women and Child Health, The Aga Khan University, Karachi, Pakistan

## Abstract

**Background:**

We aimed to describe newborn body composition and identify which anthropometric ratio (weight/length; BMI; or ponderal index, PI) best predicts fat mass (FM) and fat-free mass (FFM).

**Methods:**

Air-displacement plethysmography (PEA POD) was used to estimate FM, FFM, and body fat percentage (BF%). Associations between FFM, FM, and BF% and weight/length, BMI, and PI were evaluated in 1,019 newborns using multivariate regression analysis. Charts for FM, FFM, and BF% were generated using a prescriptive subsample (*n*=247). Standards for the best-predicting anthropometric ratio were calculated utilizing the same population used for the INTERGROWTH-21^st^ Newborn Size Standards (*n*=20,479).

**Results:**

FFM and FM increased consistently during late pregnancy. Differential FM, BF%, and FFM patterns were observed for those born preterm (34^+0^−36^+6^ weeks’ gestation) and with impaired intrauterine growth. Weight/length by gestational age (GA) was a better predictor of FFM and FM (adjusted *R*^2^=0.92 and 0.71, respectively) than BMI or PI, independent of sex, GA, and timing of measurement. Results were almost identical when only preterm newborns were studied. We present sex-specific centiles for weight/length ratio for GA.

**Conclusions:**

Weight/length best predicts newborn FFM and FM. There are differential FM, FFM, and BF% patterns by sex, GA, and size at birth.

A wide range of exposures in pregnancy can influence fetal and early postnatal growth and development. Traditionally, weight at birth has been used to quantify those intrauterine effects; however, it is increasingly acknowledged that the measure by itself, without consideration of gestational age (GA), is an inadequate predictor of health outcomes.

We have already demonstrated that preterm birth (1), newborn stunting, and wasting for GA (2) are heterogeneous syndromes, associated with different morbidities and mortality. For example, we found that wasting at birth (3) is strongly associated with duration of neonatal intensive care, respiratory distress syndrome, and no oral feeding in the first 24 h (2). However, better functional and/or tissue-specific biomarkers, such as body composition and indices of functional development, are required to improve the phenotypic characterization of newborns.

Large neonatal body composition data sets are not generally available for technical reasons; thus, mostly anthropometric indices have been used as a proxy—e.g., combinations of weight, length, skinfold thickness, and various body circumferences. Indices such as weight/length, or those in which weight is normalized with the power of length, may be practical alternatives.

The availability of air displacement plethysmography (PEA POD, Cosmed, Rome, Italy) has improved our understanding of newborn body composition and the implications of feeding regimens especially for preterm and growth-restricted newborns (4).

Here, we describe newborn air displacement plethysmography-derived data across a range of GAs from the Oxford site of the Newborn Body Composition Study (NBCS) of the INTERGROWTH-21^st^ Project, in which mothers and their babies were monitored prospectively from early pregnancy to 2 years of age. The project has generated international standards for use during pregnancy (5), as well as size of the newborn at birth for gestational age and sex (3, 6), and postnatal growth of preterm infants (7), recently recommended by WHO (8). Our aims were to evaluate newborn body composition patterns across GA and weight; determine which anthropometric index, i.e., weight/length, BMI, or ponderal index (PI), best predicts fat mass (FM) and fat-free mass (FFM); and produce prescriptive charts for FM, FFM, and body fat percentage (BF%) by GA.

## Methods

### Study Populations

The INTERGROWTH-21^st^ Project has been described elsewhere (9). Its aim was to study growth, health, nutrition, and neurodevelopment from 14 weeks’ gestation to the age of 2 years using the same conceptual framework as the WHO Multicentre Growth Reference Study (10) so as to produce prescriptive growth standards and improved perinatal phenotypes. In brief, INTERGROWTH-21^st^ was a multicenter, multicountry, population-based project. Using standardized protocols across all sites, we recruited women with reliable menstrual dates and a confirmatory ultrasound-dating scan early in pregnancy (9). We obtained institutional consent to use routinely collected data, and women gave oral consent.

The present analysis was performed in two steps with their corresponding populations: in the first step, all singleton babies, born between 7 June 2011 and 7 April 2016, to women enrolled in the INTERGROWTH-21^st^ Project at the Oxford site (the only site with a PEA POD in 2011), were eligible, provided they were alive on hospital discharge. This population constituted the NBCS (*n*=1,019) and included newborns with different intrauterine growth trajectories and anthropometric characteristics at birth to enable the relationship with body composition to be examined across a wide range of newborn nutritional states. This total NBCS population was used for the following: (i) to describe FM and FFM patterns at birth across the range of GAs; (ii) to evaluate which anthropometric index best predicted FM and FFM at birth; and (iii) to select a subpopulation, with the same prescriptive criteria used to generate the INTERGROWTH-21^st^ Standards (3, 5), to produce FM and FFM prescriptive charts (*n*=247).

In the second step, we constructed international standards for the best-predictive newborn anthropometric index (identified in the analysis described above) with the same INTERGROWTH-21^st^ population used to construct the Newborn Size Standards (3) and Very Preterm Size at Birth Reference Charts (6) (*n*=20,479).

### Newborn Anthropometric Measures

A team of anthropometrists were specially recruited, trained, and standardized for the project (11). Newborn measurements were taken within 12 h of birth using identical equipment in all sites—an electronic scale (Seca, Hamburg, Germany) for birth weight, a specially designed Harpenden infantometer for recumbent length, both calibrated twice weekly, and a metallic non-extendable tape for head circumference (Chasmors, London, UK) (12). The newborns were re-weighed at the time of PEA POD measurement, and this value was used to determine body composition.

Each measurement was collected independently and repeated by a second anthropometrist. If the difference between the two measurements exceeded 50 g (birth weight), 7 mm (length), or 5 mm (head circumference), then both observers independently took that measurement again and, if necessary, a third time. The monitoring and quality-control methods used across the sites are described in detail elsewhere (11). In the standardization sessions (Oxford site), the intra-observer error ranged from 0.118 to 0.592 cm for length, and from 0.05 to 0.435 cm for head circumference. Neonatal clinical practices, including feeding and care in a neonatal intensive care unit, were standardized for all sites (13).

### Newborn Body Composition Estimation

Body composition was estimated within 96 h of birth (to include preterm infants who were not clinically stable before, and healthy term newborns before their early discharge consistent with hospital policy) using the PEA POD; 97% were measured less than 72 h. The PEA POD is designed for use in infants up to 6 months of age or weighing up to 8 kg. It is unclear how valid estimates are at very low birth weights, but they are reliable for stable preterm babies (14).

The PEA POD estimates body composition by whole-body densitometry using a two-compartment model. Body volume is measured by air displacement plethysmography and body weight is measured using an electronic scale. A computer program calculated body composition from these data using the “Fomon” model (15). Once body volume was known and whole-body density was calculated, FM and FFM were estimated. FM density was assumed to be constant (0.9007 g/ml), whereas FFM density varies from 1.063 g/ml at birth to 1.067 g/ml at the age of 6 months (15). FM was calculated as (weight × BF%) and FFM as (weight−FM) (16). The reliability of the technique and its accuracy (17) are well established.

The PEA POD was routinely calibrated and used in a temperature-controlled room. The baby was evaluated undressed in the test chamber for 2 min, and, if necessary, duplicates of irremovable items (clamps, tubes, or tags) were measured before the examination.

### Statistical Methods

Measures were summarized according to sex, age at examination, preterm or term birth, and fetal growth by GA using means and proportions. Body composition measures approximated to a normal distribution, although the distribution of FM was slightly skewed.

Multiple linear regression analyses between weight/length ratio, BMI, and PI as the independent, explanatory variables and FM, FFM, and BF% as the dependent (outcome) variables in separate models, adjusting by sex, GA at birth (weeks), and postnatal age (hours) at body composition estimation, were carried out. The statistical methods selected to construct standards have been discussed previously (3, 10).

To estimate the standards of weight/length, fractional polynomials with two powers for the mean and one for the SD (18) were fitted separately for boys and girls. Goodness-of-fit was evaluated with visual inspection of overall model fit using quantile–quantile plots of the residuals, worm plots (19), the *Q* statistic (20), plots of residual vs. fitted values, and the distribution of fitted *z-*scores across GA.

We used STATA version 12.1 (StataCorp LP, College Station, TX, USA) or R statistical software version 3.2.4 (https://www.r-project.org), the latter using the GAMLSS framework (https://cran.r-project.org/web/packages/gamlss/index.html). Tables containing centile values and *z*-scores, and printable charts will be available free on the INTERGROWTH-21^st^ Project website (https://intergrowth21.tghn.org).

Data were entered directly into the specially developed (http://medscinet.com), online electronic data management system (http://www.medscinet.net/intergrowth/protocol.aspx?lang=1).

The INTERGROWTH-21^st^ Project was approved by the Oxfordshire Research Ethics Committee “C” (reference: 08/H0606/139), the research ethics committees of the individual participating institutions, as well as the corresponding regional health authorities where the project was implemented. A list of all study contributors is given in the Supplementary Materials online.

## Results

Results are presented following the two steps described in the Methods section. In the first step, we (i) described body composition at birth across the range of GAs (*n*=1,019); (ii) evaluated which anthropometric index best predicted FM and FFM at birth (*n*=1,019); and (iii) selected a subpopulation, with the same prescriptive criteria used to generate the INTERGROWTH-21^st^ Standards (3, 5), to produce FM and FFM prescriptive charts (*n*=247).

In the second step, we constructed international standards for the best predictive newborn anthropometric index identified in (ii) above, with the same INTERGROWTH-21^st^ population used to construct the Newborn Size Standards (3) and Very Preterm Size at Birth Reference Charts (6) (*n*=20,479).

### Body Composition at Birth

A total of 1,923 mothers, who delivered at the John Radcliffe Hospital, Oxford, in the study period and were enrolled in the INTERGROWTH-21^st^ Project, were eligible to participate in NBCS. Of their singleton babies, 219 could not be studied within the 96-h limit because of a medical condition or neonatal intensive care unit admission, 22 had very early hospital discharge, and 175 were born while the PEA POD was being serviced. In addition, 461 mothers did not wish to participate. Hence, we measured 1,046 newborns alive at discharge without congenital malformations, from which we excluded 21 measured after the 96-h limit and 6 with a FM or FFM value >3 SD.

Therefore, data from 1,019 newborns (501 boys; 518 girls) were used to describe body composition patterns and their association with perinatal conditions and anthropometric indices. The demographic and baseline characteristics of the initial 1,923 mothers, the 1,019 mothers whose babies were included in NBCS and the 461 mothers who did not wish to participate, were remarkably similar (data not shown).

In keeping with the selection criteria for the INTERGROWTH-21^st^ Project (9), women were mostly well-educated and married or cohabiting; half of the women were nulliparous (Table 1). The low number of associated morbidities and relatively low Cesarean section rate (30%) are consistent with a medium-risk population. The preterm (<37 weeks’ gestation) and term low birth weight rates were 8.9% and 4.0%, respectively. Sixty-six percent of the newborns were discharged from hospital on exclusive breastfeeding (Table 1). Individual values for weight, length, and head circumference at birth for the total study population were plotted against the sex-specific Newborn Size Standards (3) (Supplementary Figure S1), which demonstrated that the overall distribution of the total population was compatible with a population similar to that from which the Newborn Size Standards were derived.

From the 1,019 newborns measured, a subpopulation of 247 newborns (118 girls; 129 boys) met the low-risk inclusion criteria (3, 5) required to produce the NBCS prescriptive charts. Compared with the 1,019 pregnancies in the total NBCS, as expected, women in the low-risk subpopulation were healthier, thinner, and taller with very low preterm and term low birth weight rates (Table 1), confirming this as a suitable subpopulation to construct prescriptive charts.

**Figure 1:**
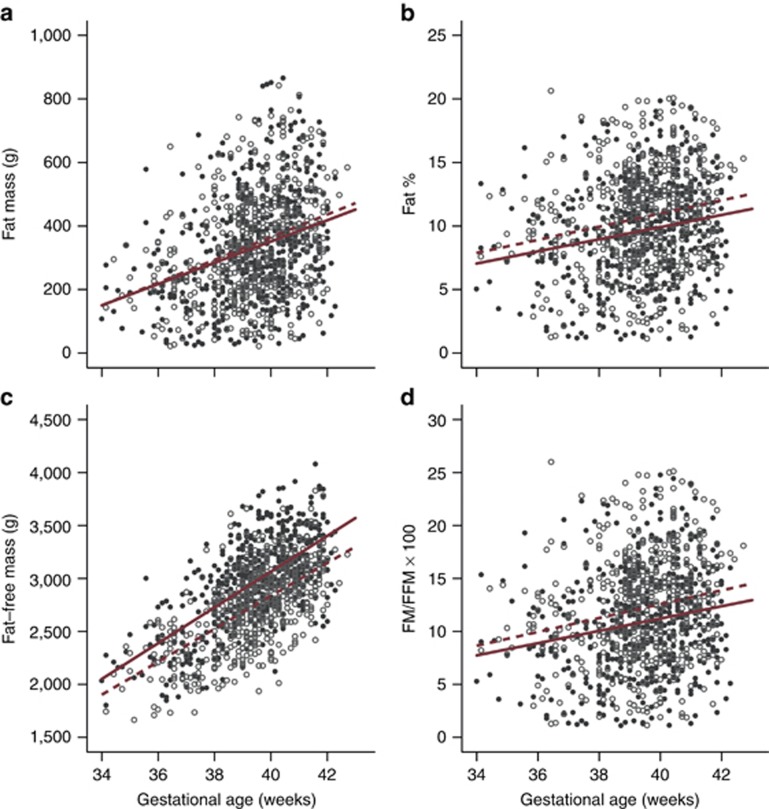
Body composition measures according to gestational age and sex. Body composition measures ((**a**) FM; (**b**) BF% (**c**) FFM; and (**d**) FM/FFM ratio) by gestational age for 501 boys (closed circles) and 518 girls (open circles) in the Newborn Body Composition Study. Superimposed is the average change per week (slope; boys, solid red lines; girls, dashed red lines). All slopes have *P* values <0.001. Differences between the sexes were significant for BF%, FFM, and FM/FFM ratio (*P*<0.001). BF%, body fat percentage; FFM, fat-free mass; FM, fat mass.

Table 2 shows detailed information about anthropometric and body composition measures, by sex, for both the total population and low-risk subpopulation. The distributions of FM, FFM, and BF% according to GA and sex are presented in Figure 1. There was a moderate increase in FM by GA with very large variability, 36 g/week (95% confidence interval (CI) 28–43 g) for girls and 33 g/week (95% CI 25–42 g) for boys. Similar patterns were observed if the data were expressed as BF% or FM/FFM ratio. Testing the regression lines at the mean GA, girls had a higher mean BF% and FM/FFM ratio than boys (*P*<0.001); however, there was no significant difference in absolute FM between the sexes (*P*=0.26).

There was a linear increase in FFM, 155 g/week (95% CI 140–171 g) for girls and 169 g/week (95% CI 152–186 g) for boys, with less variability than that for fat indicators at all GAs. Overall, average FFM increased from close to a mean of 2 kg at 34 weeks’ gestation to a mean of over 3 kg at 40 weeks’ gestation for boys and girls. FFM patterns were the inverse of those for FM, i.e., boys on average had more FFM than girls at all GAs after 34 weeks’ gestation. Testing the regression lines at the mean GA, girls had a lower mean FFM (*P*<0.001) but higher mean FM/FFM ratio (*P*=0.001). These results suggest that the increase in fetal weight late in pregnancy is mostly due to an increase in FFM in both sexes and that FFM is slightly higher in boys, whereas BF% is slightly higher in girls across GAs.

We explored the effect of postnatal age, at the time of the measurements, on weight and body composition estimates, in a cross-sectional analysis according to sex (Table 3). With increasing postnatal age (h), there were significantly lower body weights and FFMs, independent of GA at birth for both boys and girls. There were smaller nonsignificant differences in FM and BF% with postnatal age. These differences suggest that the clinically observed reduction in weight during the first 3 days of postnatal life is mostly related to FFM (Table 3).

Preterm (mostly >34 weeks’ gestation) newborns had significantly lower FM, FFM, BF%, and FM/FFM ratio than their term counterparts (Table 4). The preterms were on average 693 g lighter than the term newborns, of which 112 g (16%) was FM and 581 g (84%) was FFM; however, 112 g represented 33% of the total FM, and 581 g represented only 20% of the total FFM in the term newborns adjusted for sex and the postnatal age at the time of the PEA POD measurement.

We further explored body composition associated with small for gestational age (SGA; birth weight <10th centile), large for gestational age (LGA; birth weight>90th centile), newborn wasting (BMI<3rd centile), and stunting (length-for-GA<3rd centile), as well as late preterm birth according to the INTERGROWTH-21^st^ charts (3, 6).

Table 5 presents similar comparisons for newborns SGA and LGA vs. those with appropriate weight for gestational age (AGA). Newborns SGA had proportionally less FM, BF%, and FM/FFM ratio than newborns with AGA. Newborns SGA were on average 680 g lighter, of which 27% was FM and 73% FFM, with less than half the fat of AGA (AGA, 332 g; SGA, 140 g, i.e., 42%), but proportionally lower FFM reduction, independent of sex (AGA, 2,868 g; SGA, 2,256 g, i.e., 79%). Conversely, newborns LGA were on average 707 g heavier than newborns AGA, of which 253 g (36%) was FM and 64% FFM; proportionally, they had higher FM and less FFM (BF%: AGA, 10.2% SGA, 14.8% Table 5).

**Figure 2:**
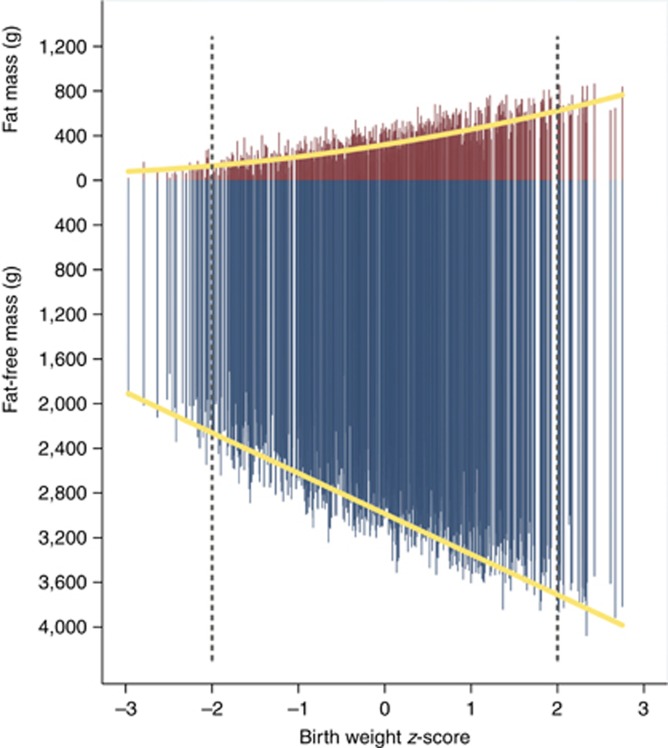
Individual measures of fat mass (red bars) and fat-free mass (blue bars) for 501 boys and 518 girls in the Newborn Body Composition Study by their birth weight *z-*scores. The dashed lines indicate 2 SDs. The mean (SD) for fat mass was 104 g (66), 335 g (158), and 670 g (110) for the <−2, −2 to 2, and >2 SD groups, respectively; mean (SD) for fat-free mass was 2,083 g (190), 2,851 g (385), and 3,633 g (233) for the <−2, −2 to 2, and >2 SD groups, respectively. Superimposed is the average change across birth weight *z-*score (yellow lines).

Figure 2 presents the simultaneous contribution of absolute FM and FFM values (as opposed to the proportional data in Table 5) across the birth weight distribution expressed as sex-specific *z*-scores (3). There appears to be a linear reduction in absolute FFM toward the lower birth weights, whereas higher birth weights involve an increase in both types of tissue.

Table 6 presents the comparisons of all stunted vs. non-stunted newborns, and wasted vs. non-wasted newborns (2). Unsurprisingly, wasted newborns had considerably lower FM values (94 vs. 346 g, 73% difference), although the FFM differences were relatively modest (2,242 vs. 2,872 g, 22% difference). On the other hand, although stunted newborns had overall lower tissue mass values, they had a smaller FM reduction of 54% compared with the non-stunted newborns with a similar FFM loss (23%).

### Prediction of Body Composition by Anthropometric Indices

Table 7 shows the relationship between the three anthropometric ratios proposed to evaluate body proportionality and FM, FFM, and BF% as outcome measures (results are expressed as *R*^2^ and regression coefficient). This analysis allowed identification of the anthropometric ratio that best predicted body composition indicators.

Multivariable linear regression analyses, adjusted by GA at birth (weeks), sex, and age at PEA POD measurement, demonstrated that the weight/length ratio by GA was the best predictor for FM and FFM as evaluated by adjusted *R*^2^ values. The simple weight/length ratio measured at birth was superior at predicting FFM than either BMI or PI (*R*^2^=0.92 vs. 0.81 and 0.62); the same pattern was also observed for FM (*R*^2^=0.71 vs. 0.64 and 0.43) and BF% (*R*^2^=0.54 vs. 0.50 and 0.35). Comparing unadjusted analyses with the adjusted data in Table 7 and stratifying these analyses by sex did not reveal any differential patterns. The results for the same analysis in the subsample of 91 preterm newborns (mostly >34 weeks’ gestation) produced almost identical values to those in Table 7 (data not shown).

We also present in Table 7 adjusted regression coefficients, expressed as g of FFM or FM per unit of difference in the corresponding anthropometric index (95% CI). Each unit difference in weight/length ratio was associated with a statistically significant higher FM of 187 g; however, differences in BMI and PI units were associated with a higher FM of only 103 and 42 g, respectively. Similarly, a unit difference in weight/length ratio was associated with 401 g higher FFM, which was considerably greater than differences of 205 and 72 g with BMI and PI units, respectively (Table 7). In summary, the weight/length ratio by GA was systematically more closely associated with the actual values of FM, FFM, and BF% than were BMI or PI at birth.

### Newborn Fat Mass, Percentage of Body Fat, and Fat-Free Mass

**Figure 3:**
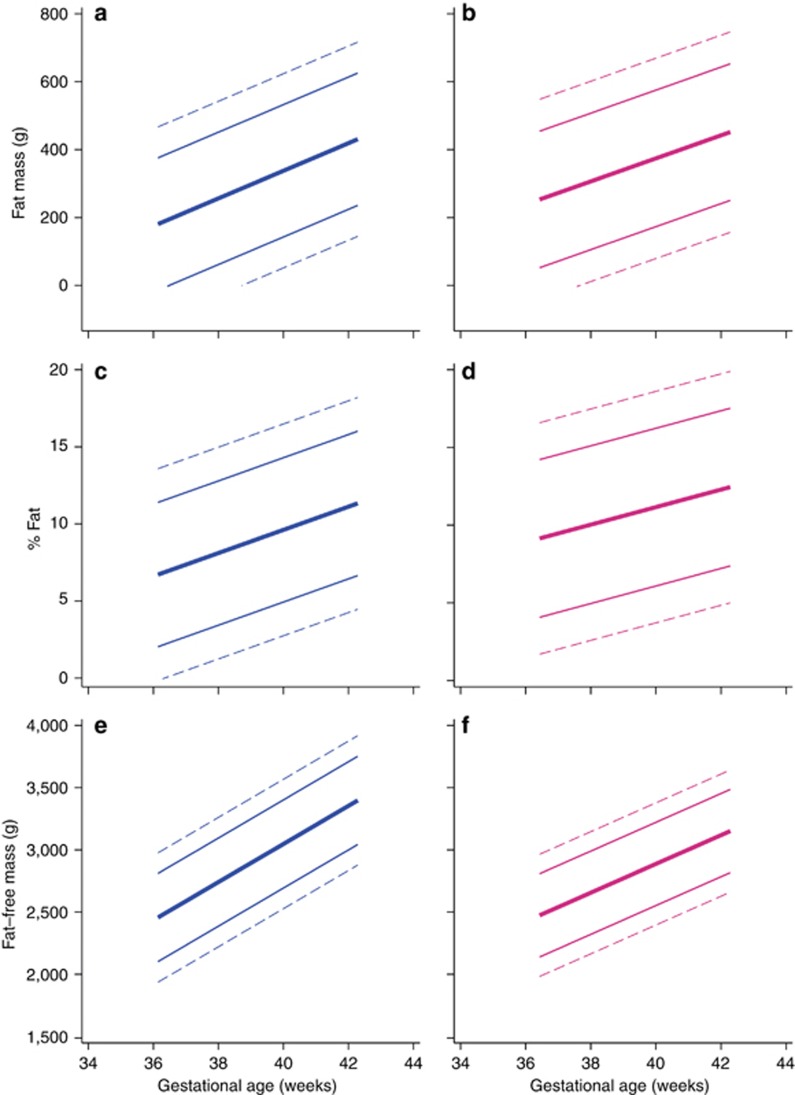
Normative centiles for body composition measures according to gestational age and sex. Centiles (3rd, 10th, 50th, 90th, and 97th) for fat mass ((**a**) boys, blue; (**b**) girls, pink); body fat percentage ((**c**) boys, blue; (**d**) girls, pink); and fat-free mass ((**e**) boys, blue; (**f**) girls, pink) according to gestational age. The data are from 129 boys and 118 girls classified as low risk (3, 5). Dashed lines below 38 weeks’ gestation should be interpreted with caution, given the small sample size.

As described above, we explored the distributions of FM, FFM, and BF%, and the corresponding indices by GA in a “prescriptive” subpopulation (*n*=247) of newborns, selected from the total NBCS population according to the same inclusion criteria used for constructing the INTERGROWTH-21^st^ standards (3, 5). For these analyses, we also excluded newborns who had one ultrasound measure *in utero* >4 SD or two or more measures >3 SD of the INTERGROWTH-21^st^ Fetal Growth Standards for GA (5). These rigorous selection criteria produced 247 newborns with normal ultrasound growth before birth (Supplementary Figure S2). Their descriptive data are shown in Table 1 and 2. Figure 3 presents the 3rd, 10th, 50th, 90th, and 97th centiles. There was very large variability in terms of FM and BF% values across this range of GAs, all compatible with healthy pregnancies, optimal fetal growth, and good neonatal outcomes. Conversely, FFM increased with GA in a more linear manner and with less variability (Figure 3) than FM. Supplementary Tables S1–S3 present the corresponding centiles for FM, FFM, and BF% according to GA.

### Newborn Weight for Length Ratio according to GA and Sex Standards

Finally, we have produced international standards for the weight/length ratio according to GA and sex using the same multicountry population (*n*=20,479) and statistical methodology as the INTERGROWTH-21^st^ Newborn Size Standards (3) and Very Preterm Size at Birth Reference Charts (6).

Comparing smoothed centiles and observed centiles, the mean differences in absolute values, independent of sex, were negligible—0.006, 0.012, and 0.08 kg/m in boys and 0.033, 0.018, and 0.068 kg/m in girls at the 3rd, 50th, and 97th centiles, respectively, for weight/length ratio. Values of smoothed centiles and observed centiles after 35 weeks’ gestation were also almost identical. The largest absolute difference was 0.85 kg/m for girls and 0.71 kg/m for boys at 35 weeks’ gestation for the 97th centile.

**Figure 4:**
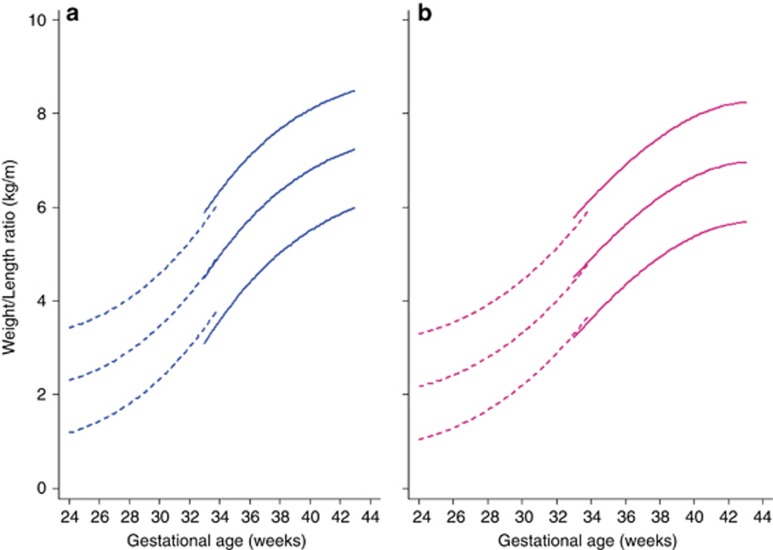
The 3rd, 50th, and 97th centiles for weight–length ratio by gestational age in the Newborn Cross-Sectional Study of the INTERGROWTH-21^st^ Project (*n*=20,479). (**a**) Boys, blue; (**b**) girls, pink. Values less than 33 weeks’ gestation are references (6) (dashed lines), followed by the INTERGROWTH-21^st^ weight–length ratio standards (3) (solid lines). Centiles below 28 weeks’ gestation should be interpreted with caution, given the small sample size.

We then estimated the 3rd, 10th, 50th, 90th, and 97th centiles according to GA and sex, which represent the international standards (⩾33 weeks’ gestation) and references (<33 weeks’ gestation) for newborn weight/length ratio (Figure 4). Supplementary Tables S4 and S5 present the corresponding values for these centiles and *z*-scores according to GA and sex. Centiles <28 weeks’ gestation should be interpreted with caution, given the small sample size.

## Discussion

We have presented a comprehensive description of newborn FM, FFM, and BF% showing the following: (i) FFM deposition has a dominant role in fetal weight changes during late pregnancy; (ii) FM and FFM patterns at birth are slightly different in boys and girls across GAs; (iii) preterm newborns have less FM, FFM, and BF% than term newborns; (iv) compared with AGA newborns, the lower birth weight of newborns SGA is related to a reduction of 27% in FM and 73% in FFM; (v) the heavier birth weight of newborns LGA consists, on average, of 36% FM and 64% FFM; (vi) the simple weight/length ratio at birth by GA is a better predictor than BMI of body composition parameters because of its stronger relationship with both BF and FFM. Accordingly, we provide international standards by GA and sex to judge the proportionality of newborn size, complementing the INTERGROWTH-21^st^ Newborn Size Standards (3, 6); finally, we offer prescriptive charts for FM, FFM, and BF%.

We have compared body composition estimates at <12, 12–24, and >24 h after birth adjusting for GA at birth. We have confirmed a pattern of weight reduction during the first 4 postnatal days of 9% (95% CI 6.6–11.2%), mostly related to loss of FFM (likely due to water loss or hydration status), independent of GA and sex, with minor or no changes in BF% (Table 3) (21). Hence, we adjusted all further analyses by the baby’s age in hours at the time of the PEA POD measurement.

Recent reviews have highlighted the difficulties of comparing estimates from available sources (22). We used air displacement plethysmography, because it is rapid, non-invasive, provides immediate results, accounts for total body water changes, is well accepted, and it has been validated for both term and preterm infants against deuterium dilution (17). Limitations include the following: (i) results are based on the estimates of Fomon *et al.* (15), which used a number of data sets and assumptions to describe a “typical” boy weighing 3.5 kg and a girl weighing 3.35 kg; (ii) it is not clear how accurate these methods are for very small preterm infants; and (iii) the newborn residual thoracic volume has to be estimated rather than measured as in adults.

Previous PEA POD studies reported average values of BF%, ranging between 7.8 and 12.3% for girls and between 7.3 and 12.3% for boys (22, 23, 24). Similarly, using magnetic resonance imaging in term AGA babies with birth weights ⩾2.5 kg, BF% values of 10.2% for boys and 10.9% for girls were reported (14).

Importantly for the external validity of our data, similar patterns were observed in a term, low-risk, Irish population (*n*=743; ref. (25)). Specifically, the 50th centiles of their BF% charts at 40 weeks’ gestation were 9.9% for boys and 12.5% for girls, very similar to our 10.0% for boys and 11.4% for girls (Figure 3 and Supplementary Table S2). BF% within the same range was also reported in a smaller study of neonatal adiposity from the United States of America (26). It is, therefore, very likely that the mean BF% at term, measured using PEA POD, for both sexes combined is 10–12%, with a SD of ~4% (24, 25, 26, 27). However, sex differences occur: girls have a higher BF% and FM/FFM ratio on average, but not FM, across GAs (14, 25).

The body composition ‘normative’ data presented here should be used cautiously because they were derived from the Oxford site, unlike other INTERGROWTH-21^st^ standards that were based on data from eight sites worldwide. It was not possible, therefore, to assess heterogeneity across sites. In addition, studying a low- to medium-risk population meant that very few early preterm births occurred despite the large sample size. Interestingly, even among low-risk newborns, the BF% values range between 3 and 20% at term, compatible with healthy neonates.

It is also important that our results should not be used to recommend postnatal feeding practices because infants were assessed before much or any postnatal feeding, and the results are derived from cross-sectional measures at birth; hence, they only demonstrate how tissues were deposited during intrauterine life. Postnatal body composition standards, produced with a similarly robust methodology, population selection, and standardized human milk-based feeding practices do not exist at present; however, a recent meta-analysis of individual cases from four studies using dual-energy X-ray absorptiometry to evaluate postnatal body composition represents a positive step toward a tool for clinical practice (28).

After 34 weeks’ gestation, we observed overall a progressive increase in FFM by GA, a greater absolute increase than that for FM and BF%, although proportionally the FM slope (average change per week) represents 10% of the mean FM, whereas the FFM slope is 6% of the total FFM (Figure 1). We also demonstrated that, at birth (Table 4), preterm babies have less BF% and FM and less FFM than their term counterparts. Low FM at birth is clinically important because both very small and late preterm babies accumulate FM by the time they reach “term”-corrected age, and so have higher BF% than term newborns (21, 28, 29, 30, 31, 32, 33). Preterm babies also have less FFM, which is relevant as reduced FFM levels remain in very preterm boys at least up to 5 years of age (34).

We described the patterns of tissue deposition *in utero* across the birth weight range in the “controlled” environment provided by relatively healthy and adequately nourished pregnant women. The data suggest that the FM and FFM dynamics are dissimilar for SGA and LGA. FFM is clearly the leading component in the process of lower or higher birth weights; conversely, expressed as BF% of term infants, the largest difference is related to FM (Table 5). However, we should consider that estimations of FFM, FM, and BF%, and their relative changes obtained from PEA POD, a two-compartment method, are not independent but complementary; they are based on FFM density assumptions (15), which could change in late gestation.

The high variability observed in the fat indicators’ data has to be interpreted, bearing in mind that, at birth, most fat is subcutaneous and very little is intra-abdominal. Furthermore, the main difference between growth-restricted and AGA newborns is in subcutaneous fat; intra-abdominal amounts are similar (14). Moreover, compared with term infants, the increase in intra-abdominal fat in healthy, preterm infants by the time they reach 40–42 weeks post-conceptual age is small (29), and preterm infants do not have a higher percentage of intra-abdominal fat by 5–7 years of age (35).

These considerations are important because the increased risks associated with adult adiposity are related to visceral, not subcutaneous, fat. It is difficult to know what role subcutaneous fat in newborns, which varies considerably in amount, has in the etiological pathway to high adult body fat and long-term health. Clearly, however, the effect on these same long-term outcomes of changes in the very small proportion of newborn intra-abdominal fat, as well as the associations with neonatal morbidities, needs to be studied, while considering that the stress associated with neonatal morbidities may itself drive the deposition of intra-abdominal fat (36).

We also demonstrated that the newborn weight/length ratio by GA and sex best reflects primarily FFM, plus FM and BF%. This accords with data from newborns and children showing that BMI is not a good indicator of body fat, but rather reflects overall body tissue (37). The reason for using length or height to a power function is the statistical effect; when a power, i.e., height^2^, is in the denominator, the association between the denominator and numerator is considerably reduced (38). The weaker association of BMI and PI with FM and FFM (Table 7) and the unknown stability of the suggested powers across populations make them less attractive for clinical and epidemiological applications (39). Hence, the weight/length standards by GA and sex presented here could be used to refine the clinical evaluation of newborns worldwide so as to facilitate comparisons across populations and possibly identify infants at risk of obesity (40).

## Figures and Tables

**Table 1:** Baseline characteristics and perinatal events of the Newborn Body Composition Study of the INTERGROWTH-21^st^ Project

	All study participants (*n*=1,019)	Low-risk pregnancies^a^ (*n*=247)
Maternal age at birth, years	31.0 (5.1)	30.2 (3.6)
Maternal height, cm	164.3 (6.8)	165.8 (6.4)
Maternal weight <15 weeks, kg	67.2 (13.9)	64.0 (9.2)
BMI <15 weeks, kg/m^2^	24.9 (4.9)	23.2 (2.9)
Paternal height^b^, cm	179.4 (6.7)	179.3 (6.7)
Gestational age at first ultrasound, weeks	12.3 (1.9)	12.2 (1.5)
Years of formal education (mother)^c^, years	15.7 (3.5)	16.3 (3.2)
Hemoglobin level at <15 weeks^d^, g/l	128.3 (9.2)	127.7 (9.1)
Married or cohabiting	981 (96.3)	243 (98.4)
Nulliparous	497 (48.8)	153 (61.9)
Preeclampsia	32 (3.1)	3 (1.2)
Pyelonephritis	5 (0.5)	1 (0.4)
Gestational diabetes mellitus	42 (4.1)	0 (0.0)
Any sexually transmitted infection	5 (0.5)	0 (0.0)
Spontaneous onset of labor	524 (51.4)	168 (68.0)
PPROM (<37 weeks)	32 (3.1)	3 (1.2)
Cesarean section	308 (30.2)	47 (19.0)
NICU stay >1 day; <3 days	26 (2.6)	3 (1.2)
Preterm (<37 weeks)	91 (8.9)	7 (2.8)
Preterm and spontaneous onset of labor	41 (4.0)	2 (0.8)
Term and LBW (<2,500 g; ⩾37 weeks)^e^	41 (4.0)	3 (1.2)
Boys	501 (49.2)	129 (52.2)
Exclusive breastfeeding at discharge	670 (65.8)	192 (77.7)

LBW, low birth weight; NICU, neonatal intensive care unit; PPROM, preterm premature rupture of membrane.

Data are mean (SD) for continuous variables or number (%) for categorical characteristics.

a Selected according to the prescriptive criteria used to generate the INTERGROWTH-21^st^ Standards (3, 5).

b Data available for 847 and 181 pregnancies.

c Data available for 1,916 and 247 pregnancies.

d Data available for 1,849 and 241 pregnancies.

e Low-birth weight newborns among those born at term.

**Table 2:** Anthropometric and body composition measures of newborns enrolled in the Newborn Body Composition Study of the INTERGROWTH-21^st^ Project

	Boys (*n*=501)	Girls (*n*=518)	Total (*n*=1,019)	Low-risk newborns^a^ (*n*=247)
Age at examination, h	20.8 (16.3)	19.3 (15.2)	20.0 (15.8)	17.8 (12.9)
Gestational age at delivery, weeks	39.4 (1.6)	39.4 (1.5)	39.4 (1.6)	40.0 (1.3)
Birth weight, g	3,349 (549)	3,129 (514)	3,237 (542)	3,372 (453)
Birth weight *z*-score^b^	0.1 (1.0)	−0.1 (1.1)	0.0 (1.1)	0.1 (0.9)
Birth length, cm	49.3 (2.3)	48.2 (2.2)	48.8 (2.3)	49.6 (2.0)
Birth length *z*-score^b^	−0.1 (1.1)	−0.3 (1.1)	−0.2 (1.1)	0.1 (1.0)
Head circumference, cm	34.5 (1.4)	33.6 (1.4)	34.0 (1.4)	34.3 (1.2)
Head circumference *z*-score^b^	0.3 (1.0)	0.1 (1.0)	0.2 (1.1)	0.3 (0.9)
Weight to length ratio, kg/m	6.7 (0.9)	6.4 (0.8)	6.5 (0.9)	6.7 (0.7)
BMI, kg/m^2^	13.5 (1.3)	13.2 (1.4)	13.3 (1.4)	13.4 (1.2)
Ponderal index, kg/m^3^	27.3 (2.3)	27.2 (2.4)	27.3 (2.4)	27.1 (2.3)
Fat mass, g	332 (172)	343 (167)	337 (170)	355 (165)
Percentage body fat, %	9.6 (4.0)	10.7 (4.0)	10.2 (4.0)	10.3 (3.9)
Fat-free mass, g	2,965 (422)	2,739 (390)	2,850 (421)	2,968 (336)

Data are mean (SD) for continuous variables or number (%) for categorical characteristics.

a Selected according to the prescriptive criteria used to generate the INTERGROWTH-21^st^ Standards (3, 5).

b Calculated for each newborn using the INTERGROWTH-21^st^ Newborn Size Standards (3).

**Table 3:** Body composition measures according to age at examination of newborns enrolled in the Newborn Body Composition Study of the INTERGROWTH-21^st^ Project

	Age at examination (h)	*N*	Weight at examination (g)	*P*	Fat mass (g)	*P*	Fat percentage (%)	*P*	Fat-free mass (g)	*P*
Boys (*n*=501)	<12	179	3,439 (486)	—	350 (166)	—	9.9 (3.7)	—	3,089 (374)	—
	12–24	166	3,279 (550)	<0.01	327 (173)	0.22	9.5 (4.1)	0.42	2,952 (421)	<0.01
	>24 to <96	156	3,151 (570)	<0.01	315 (175)	0.10	9.5 (4.2)	0.47	2,836 (437)	<0.01
Girls (*n*=518)	<12	190	3,202 (488)	—	364 (156)	—	11.0 (3.6)	—	2,839 (372)	—
	12–24	188	3,114 (508)	0.02	353 (176)	0.43	10.8 (4.3)	0.67	2,761 (375)	<0.01
	>24 to <96	140	2,874 (508)	<0.01	300 (165)	0.01	9.9 (4.2)	0.08	2,574 (382)	<0.01

Data are mean (SD).

*P* values correspond to the mean differences comparing to the <12-h category; adjusted by gestational age (weeks).

**Table 4:** Body composition measures for term and preterm newborns in the Newborn Body Composition Study of the INTERGROWTH-21^st^ Project

	Preterm (*n*=91)	Term (*n*=928)	Adjusted mean difference^a^ (95% CI)
Gestational age, weeks	36.0 (0.7)	39.7 (1.2)	−3.7 (−3.8, −3.5)
Weight at examination, g	2,501 (369)	3,254 (509)	−693 (−777, −610)
Fat mass, g	226 (124)	348 (170)	−112 (−141, −84)
Percentage body fat, %	8.6 (3.9)	10.3 (4.0)	−1.5 (−2.3, −0.6)
Fat-free mass, g	2,275 (278)	2,906 (389)	−581 (−644, −519)
FM/FFM × 100	9.7 (4.7)	11.7 (5.0)	−1.9 (−2.9, −0.8)

CI, confidence interval; FFM, fat-free mass; FM, fat mass.

Data are mean (SD).

Preterm birth: <37 weeks’ gestation.

a Mean difference between categories; adjusted by sex and age of examination (hours).

**Table 5:** Body composition measures of newborns classified as small, appropriate, and large for gestational age in the Newborn Body Composition Study of the INTERGROWTH-21^st^ Project

	Small for gestational age (*n*=139)	Appropriate for gestational age (*n*=760)	Large for gestational age (*n*=120)	Adjusted mean difference SGA–AGA^a^ (95% CI)	Adjusted mean difference LGA–AGA^a^ (95% CI)
Gestational age, weeks	38.9 (1.6)	39.4 (1.6)	39.9 (1.2)	−0.5 (−0.8, −0.2)	0.5 (0.3, 0.8)
Weight at examination, g	2,404 (276)	3,201 (382)	4,007 (310)	−680 (−710, −650)	707 (660, 755)
Fat mass, g	140 (80)	332 (131)	596 (126)	−184 (−200, −168)	253 (229, 277)
Percentage body fat, %	5.7 (3.0)	10.2 (3.5)	14.8 (2.8)	−4.6 (−5.1, −4.0)	4.6 (4.0, 5.1)
Fat-free mass, g	2,265 (249)	2,868 (321)	3,411 (265)	−496 (−522, −470)	455 (416, 493)
FM/FFM × 100	6.2 (3.5)	11.6 (4.3)	17.5 (3.8)	−5.5 (−6.1, −4.8)	5.9 (5.2, 6.7)

AGA, appropriate for gestational age (3); CI, confidence interval; FFM, fat-free mass; FM, fat mass; LGA, large for gestational age, defined as >90th centile of birth weight for gestational age (3); SGA, small for gestational age, defined as <10th centile of birth weight for gestational age (3).

Data are mean (SD).

a Mean difference adjusted by sex, age of examination (hours), and gestational age at birth (weeks; except for gestational age).

**Table 6:** Body composition measures of newborns classified as wasted or stunted in the Newborn Body Composition Study of the INTERGROWTH-21^st^ Project

	Wasting			Stunting		
	Yes (*n*=35)	No (*n*=984)	Adjusted mean difference^a^ (95% CI)	Yes (*n*=63)	No (*n*=956)	Adjusted mean difference^a^ (95% CI)
Gestational age, weeks	39.1 (1.5)	39.4 (1.6)	−0.2 (−0.7, 0.3)	38.8 (1.6)	39.4 (1.6)	−0.6 (−1.0, −0.2)
Weight at examination, g	2,337 (330)	3,217 (523)	−756 (−828, −685)	2,391 (321)	3,240 (512)	−698 (−756, −641)
Fat mass, g	94 (58)	346 (166)	−239 (−265, −213)	159 (98)	349 (167)	−172 (−196, −148)
Percentage body fat, %	4.0 (2.5)	10.4 (3.9)	−6.3 (−7.2, −5.4)	6.4 (3.4)	10.4 (4.0)	−3.9 (−4.7, −3.1)
Fat-free mass, g	2,242 (321)	2,872 (408)	−517 (−583, −452)	2,232 (263)	2,891 (397)	−526 (−572, −480)
FM/FFM × 100	4.3 (2.8)	11.8 (4.9)	−7.5 (−8.5, −6.5)	7.0 (4.0)	11.8 (5.0)	−4.7 (−5.7, −3.7)

CI, confidence interval; FFM, fat-free mass; FM, fat mass.

Data are mean (SD).

Wasting: <3rd centile of body mass index for gestational age (2). Stunting: <3rd centile of birth length for gestational age (2).

A newborn could be classified simultaneously as wasted and stunted.

a Mean difference between categories adjusted by sex, age of examination (hours), and gestational age at birth (weeks; except for gestational age).

**Table 7:** Relationship between body composition measures and anthropometric ratios for 1,019 newborns enrolled in the Newborn Body Composition Study of the INTERGROWTH-21^st^ Project

	Fat mass (g)		Fat percentage (%)		Fat-free mass (g)	
	*β* (95% CI)	*R*^2^	*β* (95% CI)	*R*^2^	*β* (95% CI)	*R*^2^
Weight–length ratio, kg/m	187 (180, 195)	0.71	4.0 (3.8, 4.2)	0.54	401 (390, 412)	0.92
Body mass index, kg/m^2^	103 (98, 108)	0.64	2.3 (2.2, 2.4)	0.50	205 (195, 214)	0.81
Ponderal index, kg/m^3^	42 (39, 46)	0.43	1.0 (0.9, 1.1)	0.35	72 (66, 79)	0.62

*β*, beta coefficients from linear regression models adjusted by gestational age at delivery (weeks), age at examination (hours), and sex, with robust SEs; CI, confidence interval; *R*^2^, adjusted *R* squared.
