# Supplementary material for: Body composition at birth and its relationship with neonatal anthropometric ratios: the newborn body composition study of the INTERGROWTH-21st project
Source: Pediatr Res. 2017 May 31;82(2):305–16. doi: 10.1038/pr.2017.52 (PMC5605677; doi:10.1038/pr.2017.52)
Supplement: Supplementary Information [file pr201752x1.docx]

**Author Contributions**

Professors Villar and Kennedy had full access to all of the data in the study and take responsibility for the integrity of the data and the accuracy of the data analysis. Professors Villar, Kennedy and Bhutta were joint senior authors.

*Study concept and design:* Villar, Cheikh Ismail, Puglia, Giuliani, Kennedy.

*Acquisition, analysis, or interpretation of data:* Puglia, Victora, Fenton, Pang, Giuliani, Barros, Ochieng, Jaffer, Gravett, Purwar, Frederick.

*Drafting of the manuscript:* Villar, Kennedy, Victora, Fenton, Puglia.

*Critical revision of the manuscript for important intellectual content:* Puglia, Giuliani, Cheikh Ismail, Fenton, Victora, Uauy

*Statistical analysis:* Staines-Urias, Ohuma, Victora, Fenton, Altman.

*Obtained funding:* Villar, Kennedy.

*Administrative, technical, or material support:* Sullivan, Lambert, Papageorghiou, Noble, Bhutta

*Study supervision: Cheikh Ismail, Villar.*

**Group Information: Members of the International Fetal and Newborn Growth Consortium for the 21^st^ Century (INTERGROWTH-21^st^) and its Committees**

**Scientific Advisory Committee**

M Katz (Chair from January 2011), MK Bhan, C Garza, S Zaidi, A Langer, PM Rothwell (from February 2011), Sir D Weatherall (Chair until December 2010).

**Steering Committee**

ZA Bhutta (Chair), J Villar (Principal Investigator), S Kennedy (Project Director), DG Altman, FC Barros, E Bertino, F Burton, M Carvalho, L Cheikh Ismail, WC Chumlea, MG Gravett, YA Jaffer, A Lambert, P Lumbiganon, JA Noble, RY Pang, AT Papageorghiou, M Purwar, J Rivera, C Victora.

**Executive Committee**

J Villar (Chair), DG Altman, ZA Bhutta, L Cheikh Ismail, S Kennedy, A Lambert, JA Noble, AT Papageorghiou.

**Project Coordinating Unit**

J Villar (Head), S Kennedy, L Cheikh Ismail, A Lambert, AT Papageorghiou, M Shorten, L Hoch (until May 2011), HE Knight (until August 2011), EO Ohuma (from September 2010), C Cosgrove (from July 2011), I Blakey (from March 2011).

**Data Analysis Group**

DG Altman (Head), EO Ohuma, J Villar.

**Data Management Group**

DG Altman (Head), F Roseman, N Kunnawar, SH Gu, JH Wang, MH Wu, M Domingues, P Gilli, L Juodvirsiene, L Hoch (until May 2011), N Musee (until June 2011), H Al-Jabri (until October 2010), S Waller (until June 2011), C Cosgrove (from July 2011), D Muninzwa (from October 2011), EO Ohuma (from September 2010), D Yellappan (from November 2010), A Carter (from July 2011), D Reade (from June 2012), R Miller (from June 2012).

**Ultrasound Group**

AT Papageorghiou (Head), L Salomon (Senior external advisor), A Leston, A Mitidieri, F Al-Aamri, W Paulsene, J Sande, WKS Al-Zadjali, C Batiuk, S Bornemeier, M Carvalho, M Dighe, P Gaglioti, N Jacinta, S Jaiswal, JA Noble, K Oas, M Oberto, E Olearo, MG Owende, J Shah, S Sohoni, T Todros, M Venkataraman, S Vinayak, L Wang, D Wilson, QQ Wu, S Zaidi, Y Zhang, P Chamberlain (until September 2012), D Danelon (until July 2010), I Sarris (until June 2010), J Dhami (until July 2011), C Ioannou (until February 2012), CL Knight (from October 2010), R Napolitano (from July 2011), S Wanyonyi (from May 2012), C Pace (from January 2011), V Mkrtychyan (from June 2012).

**Anthropometry Group**

L Cheikh Ismail (Head), WC Chumlea (Senior external advisor), F Al-Habsi, ZA Bhutta, A Carter, M Alija, JM Jimenez-Bustos, J Kizidio, F Puglia, N Kunnawar, H Liu, S Lloyd, D Mota, R Ochieng, C Rossi, M Sanchez Luna, YJ Shen, HE Knight (until August 2011), DA Rocco (from June 2012), IO Frederick (from June 2012).

**Neonatal Group**

ZA Bhutta (Head), E Albernaz, M Batra, BA Bhat, E Bertino, P Di Nicola, F Giuliani, I Rovelli, K McCormick, R Ochieng, RY Pang, V Paul, V Rajan, A Wilkinson, A Varalda (from September 2012).

**Environmental Health Group**

B Eskenazi (Head), LA Corra, H Dolk, J Golding, A Matijasevich, T de Wet, JJ Zhang, A Bradman, D Finkton, O Burnham, F Farhi.

**Participating countries and local investigators**

*Brazil:* FC Barros (Principal Investigator), M Domingues, S Fonseca, A Leston, A Mitidieri, D Mota, IK Sclowitz, MF da Silveira.

*China:* RY Pang (Principal Investigator), YP He, Y Pan, YJ Shen, MH Wu, QQ Wu, JH Wang, Y Yuan, Y Zhang.

*India:* M Purwar (Principal Investigator), A Choudhary, S Choudhary, S Deshmukh, D Dongaonkar, M Ketkar, V Khedikar, N Kunnawar, C Mahorkar, I Mulik, K Saboo, C Shembekar, A Singh, V Taori, K Tayade, A Somani.

*Italy:* E Bertino (Principal Investigator), P Di Nicola, M Frigerio, G Gilli, P Gilli, M Giolito, F Giuliani, M Oberto, L Occhi, C Rossi, I Rovelli, F Signorile, T Todros.

*Kenya:* W Stones and M Carvalho (Co- Principal Investigators), J Kizidio, R Ochieng, J Shah, , S Vinayak, N Musee (until June 2011), C Kisiang’ani (until July 2011), D Muninzwa (from August 2011).

*Oman:* YA Jaffer (Principal Investigator), J Al-Abri, J Al-Abduwani, FM Al-Habsi, H Al-Lawatiya, B Al-Rashidiya, WKS Al-Zadjali, FR Juangco, M Venkataraman, H Al-Jabri (until October 2010), D Yellappan (from November 2010).

*UK:* S Kennedy (Principal Investigator), L Cheikh Ismail, AT Papageorghiou, F Roseman, A Lambert, EO Ohuma, S Lloyd, R Napolitano (from July 2011), C Ioannou (until February 2012), I Sarris (until June 2010).

*USA:* MG Gravett (Principal Investigator), C Batiuk, M Batra, S Bornemeier, M Dighe, K Oas, W Paulsene, D Wilson, IO Frederick, HF Andersen, SE Abbott, AA Carter, H Algren, DA Rocco, TK Sorensen, D Enquobahrie, S Waller (until June 2011).

**Additional Contributions**

We would like to thank the Health Authorities in Pelotas, Brazil; Beijing, China; Nagpur, India; Turin, Italy; Nairobi, Kenya; Muscat, Oman; Oxford, UK and Seattle, USA, who facilitated the project by allowing participation of these study sites as collaborating centers. We are extremely grateful to Philips Medical Systems who provided the ultrasound equipment and technical assistance throughout the project. We also thank MedSciNet U.K. Ltd for setting up the INTERGROWTH-21^st^ web-site and for the development, maintenance and support of the on-line data management system.

We thank the parents and infants who participated in the studies and the more than 200 members of the research teams who made the implementation of this project possible. The participating hospitals included: Brazil, Pelotas (Hospital Miguel Piltcher, Hospital São Francisco de Paula, Santa Casa de Misericórdia de Pelotas, and Hospital Escola da Universidade Federal de Pelotas); China, Beijing (Beijing Obstetrics & Gynecology Hospital, Shunyi Maternal & Child Health Centre, and Shunyi General Hospital); India, Nagpur (Ketkar Hospital, Avanti Institute of Cardiology Private Limited, Avantika Hospital, Gurukrupa Maternity Hospital, Mulik Hospital & Research Centre, Nandlok Hospital, Om Women’s Hospital, Renuka Hospital & Maternity Home, Saboo Hospital, Brajmonhan Taori Memorial Hospital, and Somani Nursing Home); Kenya, Nairobi (Aga Khan University Hospital, MP Shah Hospital and Avenue Hospital); Italy, Turin (Ospedale Infantile Regina Margherita Sant’ Anna and Azienda Ospedaliera Ordine Mauriziano); Oman, Muscat (Khoula Hospital, Royal Hospital, Wattayah Obstetrics & Gynaecology Poly Clinic, Wattayah Health Centre, Ruwi Health Centre, Al-Ghoubra Health Centre and Al-Khuwair Health Centre); UK, Oxford (John Radcliffe Hospital) and USA, Seattle (University of Washington Hospital, Swedish Hospital, and Providence Everett Hospital).

Full acknowledgement of all those who contributed to the development of the INTERGROWTH-21^st^ Project protocol appears at [www.intergrowth21.org.uk](http://www.intergrowth21.org.uk)
