# Supplementary material for: Body composition at birth and its relationship with neonatal anthropometric ratios: the newborn body composition study of the INTERGROWTH-21st project
Source: Pediatr Res. 2017 May 31;82(2):305–16. doi: 10.1038/pr.2017.52 (PMC5605677; doi:10.1038/pr.2017.52)
Supplement: Supplementary Figure 1 [file pr201752x2.docx]

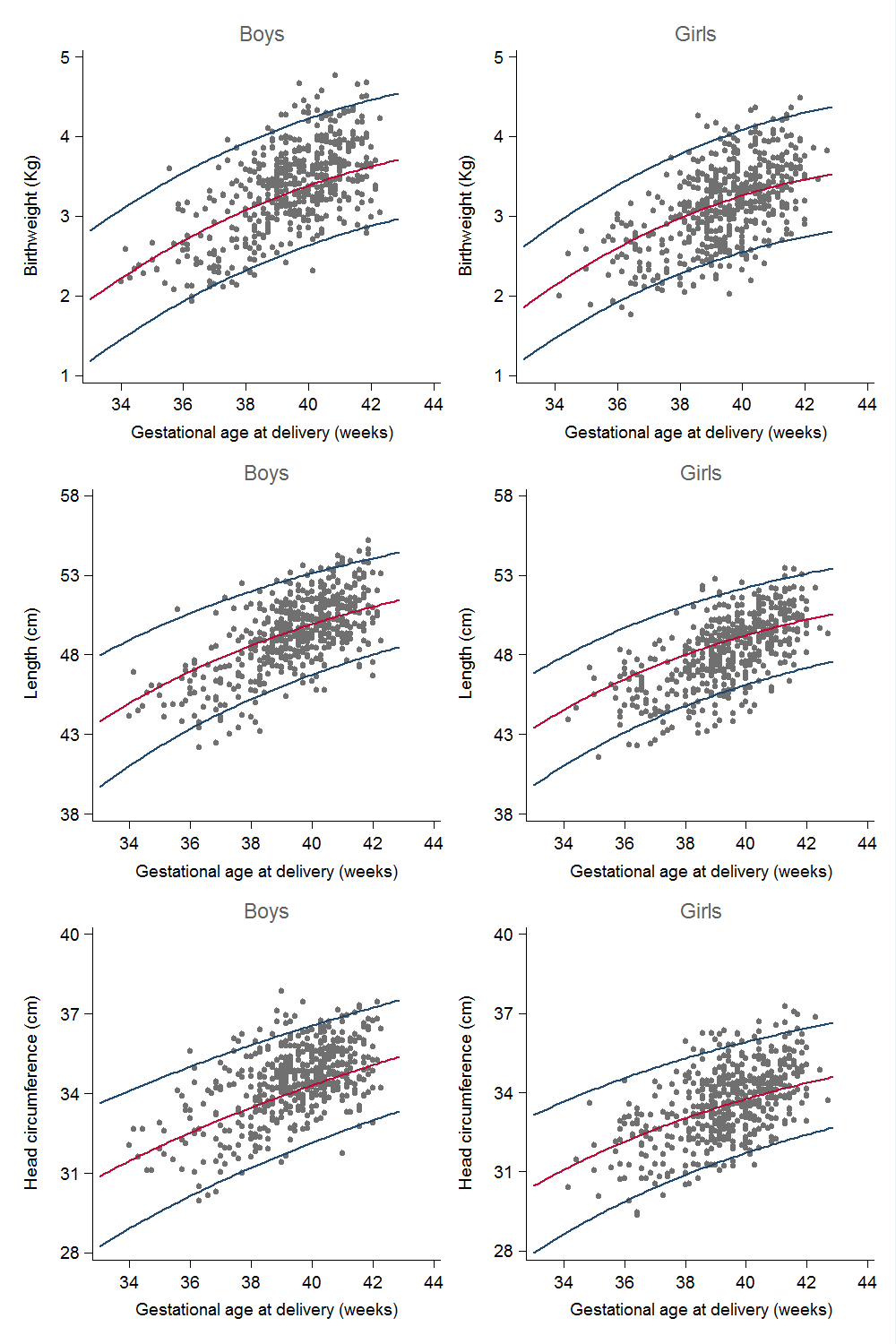


**Figure S1.** Anthropometric measures at birth by gestational age for 501 boys and 518 girls of the Newborn Body Composition Study. Superimposed are the 3^rd^, 50^th^ and 97^th^ smoothed INTERGROWTH-21^st^ standard centile curves for birthweight, birth length and head circumference according to gestational age (3).
