# Supplementary material for: Body composition at birth and its relationship with neonatal anthropometric ratios: the newborn body composition study of the INTERGROWTH-21st project
Source: Pediatr Res. 2017 May 31;82(2):305–16. doi: 10.1038/pr.2017.52 (PMC5605677; doi:10.1038/pr.2017.52)
Supplement: Supplementary Figure 2 [file pr201752x3.docx]

**
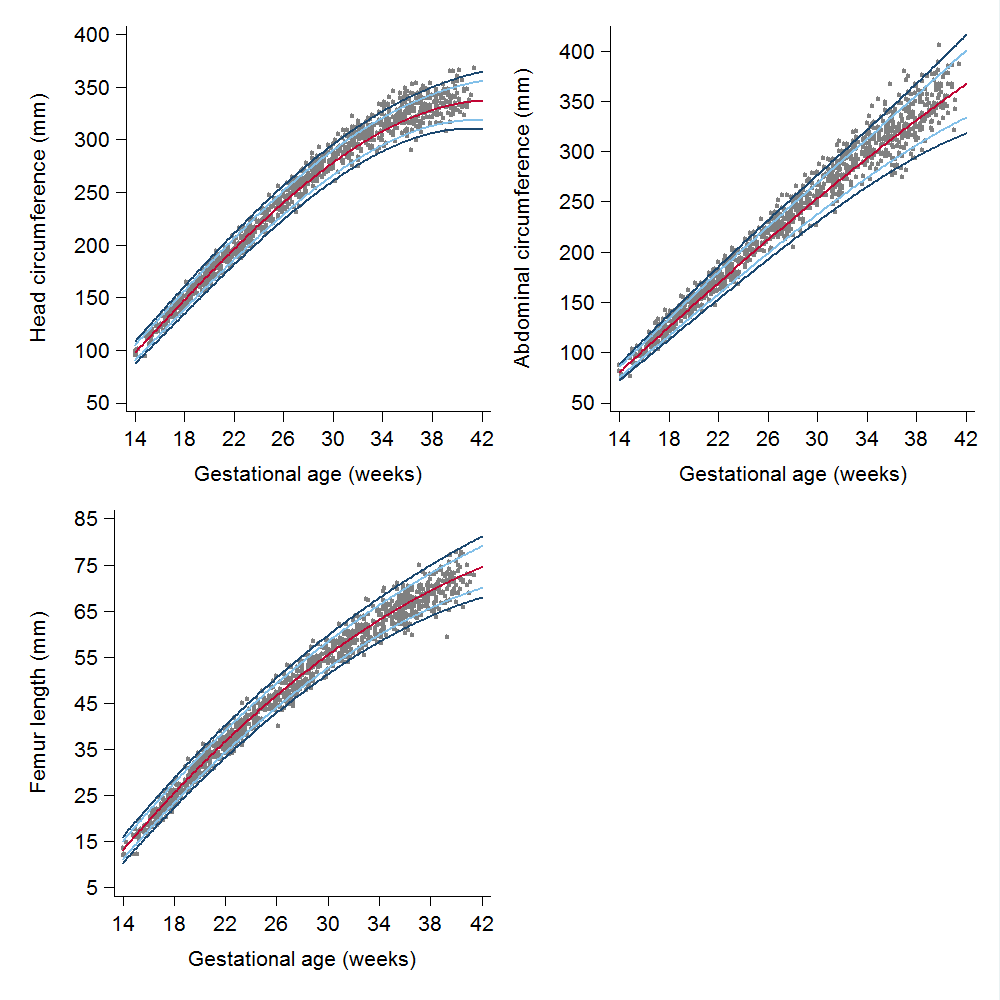
**

**Figure S2.** Longitudinal ultrasound measurements of head circumference, abdominal circumference and femur length for 129 boys and 118 girls classified as low risk in the Newborn Body Composition Study. Superimposed are the 3^rd^, 10^th^, 50^th^, 90^th^ and 97^th^ smoothed Fetal Growth Longitudinal Study standard centile curves for the corresponding ultrasound measure according to gestational age (5).
