# Supplementary material for: Body composition at birth and its relationship with neonatal anthropometric ratios: the newborn body composition study of the INTERGROWTH-21st project
Source: Pediatr Res. 2017 May 31;82(2):305–16. doi: 10.1038/pr.2017.52 (PMC5605677; doi:10.1038/pr.2017.52)
Supplement: Supplementary Table 1 [file pr201752x4.docx]

| **Table S1. Centiles for fat mass according to gestational age for low-risk newborns enrolled in the Newborn Body Composition Study of the INTERGROWTH-21^st^ Project** | | | | | | | | | | | | |
| --- | --- | --- | --- | --- | --- | --- | --- | --- | --- | --- | --- | --- |
| Gestational age at birth | Centiles for fat mass (g) | | | | | | | | | | | |
|  | Number of observations | Boys | | | | | Number of observations | Girls | | | | |
|  |  | 3^rd^ | 10^th^ | 50^th^ | 90^th^ | 97^th^ |  | 3^rd^ | 10^th^ | 50^th^ | 90^th^ | 97^th^ |
| 38 weeks | 18 | - | 83 | 277 | 472 | 563 | 15 | 29 | 123 | 324 | 525 | 619 |
| 39 weeks | 31 | 30 | 121 | 316 | 511 | 602 | 33 | 62 | 156 | 357 | 558 | 652 |
| 40 weeks | 39 | 70 | 161 | 356 | 551 | 642 | 35 | 96 | 190 | 391 | 592 | 686 |
| 41 weeks | 24 | 112 | 203 | 398 | 593 | 684 | 26 | 127 | 221 | 422 | 623 | 717 |
| 42 weeks | 7 | 136 | 227 | 422 | 617 | 708 | 3 | 150 | 244 | 445 | 646 | 740 |
| Total | 119 |  |  |  |  |  | 112 |  |  |  |  |  |
| Centiles were truncated below 38 weeks given the small sample size, but curves were constructed using all available data (n=247). | | | | | | | | | | | | |

| **Table S2. Centiles for body fat percentage according to gestational age for low-risk newborns enrolled in the Newborn Body Composition Study of the INTERGROWTH-21^st^ Project** | | | | | | | | | | | | |
| --- | --- | --- | --- | --- | --- | --- | --- | --- | --- | --- | --- | --- |
| Gestational age at birth | Centiles for body fat percentage (%) | | | | | | | | | | | |
|  | Number of observations | Boys | | | | | Number of observations | Girls | | | | |
|  |  | 3^rd^ | 10^th^ | 50^th^ | 90^th^ | 97^th^ |  | 3^rd^ | 10^th^ | 50^th^ | 90^th^ | 97^th^ |
| 38 weeks | 18 | 1.6 | 3.8 | 8.5 | 13.2 | 15.4 | 15 | 2.9 | 5.2 | 10.3 | 15.4 | 17.8 |
| 39 weeks | 31 | 2.3 | 4.5 | 9.2 | 13.9 | 16.1 | 33 | 3.4 | 5.8 | 10.9 | 15.9 | 18.3 |
| 40 weeks | 39 | 3.1 | 5.3 | 10.0 | 14.6 | 16.8 | 35 | 4.0 | 6.4 | 11.4 | 16.5 | 18.9 |
| 41 weeks | 24 | 3.9 | 6.0 | 10.7 | 15.4 | 17.6 | 26 | 4.5 | 6.9 | 12.0 | 17.0 | 19.4 |
| 42 weeks | 7 | 4.3 | 6.5 | 11.2 | 15.9 | 18.0 | 3 | 4.9 | 7.3 | 12.3 | 17.4 | 19.8 |
| Total | 119 |  |  |  |  |  | 112 |  |  |  |  |  |
| Centiles were truncated below 38 weeks given the small sample size, but curves were constructed using all available data (n=247). | | | | | | | | | | | | |

| **Table S3: Centiles for fat-free mass according to gestational age for low-risk newborns enrolled in the Newborn Body Composition Study of the INTERGROWTH-21^st^ Project** | | | | | | | | | | | | |
| --- | --- | --- | --- | --- | --- | --- | --- | --- | --- | --- | --- | --- |
| Gestational age at birth  (weeks) | Centiles for fat-free mass (g) | | | | | | | | | | | |
|  | Number of observations | Boys | | | | | Number of observations | Girls | | | | |
|  |  | 3^rd^ | 10^th^ | 50^th^ | 90^th^ | 97^th^ |  | 3^rd^ | 10^th^ | 50^th^ | 90^th^ | 97^th^ |
| 38 weeks | 18 | 2300 | 2465 | 2820 | 3174 | 3340 | 15 | 2223 | 2379 | 2714 | 3048 | 3205 |
| 39 weeks | 31 | 2446 | 2612 | 2966 | 3320 | 3486 | 33 | 2336 | 2493 | 2827 | 3162 | 3318 |
| 40 weeks | 39 | 2597 | 2762 | 3117 | 3471 | 3637 | 35 | 2454 | 2610 | 2945 | 3279 | 3436 |
| 41 weeks | 24 | 2753 | 2919 | 3273 | 3628 | 3793 | 26 | 2558 | 2715 | 3050 | 3384 | 3541 |
| 42 weeks | 7 | 2846 | 3012 | 3366 | 3721 | 3886 | 3 | 2638 | 2795 | 3129 | 3464 | 3620 |
| Total | 119 |  |  |  |  |  | 112 |  |  |  |  |  |
| Centiles were truncated below 38 weeks given the small sample size, but curves were constructed using all available data (n=247). | | | | | | | | | | | | |

| **Table S4: Centiles for weight-length ratio according to gestational age of newborns enrolled in the Newborn Cross-Sectional Study of the INTERGROWTH-21^st^ Project (3, 6)** | | | | | | | | | | | | | |
| --- | --- | --- | --- | --- | --- | --- | --- | --- | --- | --- | --- | --- | --- |
| Gestational age at birth  (weeks) | Centiles for weight-for-length ratio (Kg/m) | | | | | | | | | | | | |
|  | Number of observations | Boys | | | | | Number of observations | Girls | | | | | |
|  |  | 3^rd^ | 10^th^ | 50^th^ | 90^th^ | 97^th^ |  | 3^rd^ | 10^th^ | 50^th^ | 90^th^ | 97^th^ |  |
| 24 weeks | 6 | 1.18 | 1.54 | 2.31 | 3.07 | 3.43 | 8 | 1.04 | 1.40 | 2.17 | 2.93 | 3.29 |  |
| 25 weeks | 13 | 1.29 | 1.65 | 2.41 | 3.18 | 3.54 | 10 | 1.15 | 1.51 | 2.28 | 3.04 | 3.40 |  |
| 26 weeks | 15 | 1.43 | 1.79 | 2.55 | 3.32 | 3.68 | 9 | 1.29 | 1.65 | 2.41 | 3.18 | 3.54 |  |
| 27 weeks | 14 | 1.60 | 1.96 | 2.72 | 3.49 | 3.85 | 12 | 1.46 | 1.82 | 2.59 | 3.35 | 3.71 |  |
| 28 weeks | 22 | 1.81 | 2.16 | 2.93 | 3.70 | 4.05 | 19 | 1.67 | 2.03 | 2.79 | 3.56 | 3.91 |  |
| 29 weeks | 22 | 2.05 | 2.41 | 3.17 | 3.94 | 4.30 | 24 | 1.91 | 2.27 | 3.03 | 3.80 | 4.16 |  |
| 30 weeks | 28 | 2.33 | 2.69 | 3.45 | 4.22 | 4.58 | 29 | 2.19 | 2.55 | 3.31 | 4.08 | 4.44 |  |
| 31 weeks | 41 | 2.65 | 3.01 | 3.77 | 4.54 | 4.90 | 35 | 2.51 | 2.87 | 3.64 | 4.40 | 4.76 |  |
| 32 weeks | 53 | 3.02 | 3.37 | 4.14 | 4.90 | 5.26 | 48 | 2.88 | 3.24 | 4.00 | 4.77 | 5.12 |  |
| 33 weeks | 33 | 3.09 | 3.53 | 4.48 | 5.44 | 5.88 | 17 | 3.22 | 3.62 | 4.49 | 5.37 | 5.77 |  |
| 34 weeks | 48 | 3.58 | 4.02 | 4.96 | 5.90 | 6.34 | 65 | 3.61 | 4.02 | 4.89 | 5.76 | 6.17 |  |
| 35 weeks | 128 | 4.01 | 4.45 | 5.38 | 6.31 | 6.75 | 111 | 3.99 | 4.40 | 5.27 | 6.14 | 6.55 |  |
| 36 weeks | 320 | 4.39 | 4.82 | 5.74 | 6.66 | 7.10 | 292 | 4.34 | 4.74 | 5.62 | 6.49 | 6.90 |  |
| 37 weeks | 849 | 4.73 | 5.15 | 6.06 | 6.97 | 7.40 | 799 | 4.65 | 5.06 | 5.93 | 6.80 | 7.21 |  |
| 38 weeks | 2,031 | 5.02 | 5.44 | 6.34 | 7.24 | 7.66 | 1,786 | 4.93 | 5.34 | 6.21 | 7.08 | 7.49 |  |
| 39 weeks | 2,983 | 5.27 | 5.69 | 6.58 | 7.47 | 7.89 | 2,846 | 5.17 | 5.58 | 6.45 | 7.32 | 7.73 |  |
| 40 weeks | 2,531 | 5.50 | 5.91 | 6.79 | 7.67 | 8.08 | 2,486 | 5.37 | 5.78 | 6.65 | 7.52 | 7.93 |  |
| 41 weeks | 1,146 | 5.69 | 6.10 | 6.96 | 7.83 | 8.24 | 1,180 | 5.53 | 5.93 | 6.80 | 7.68 | 8.08 |  |
| 42 weeks | 202 | 5.85 | 6.26 | 7.11 | 7.97 | 8.37 | 218 | 5.63 | 6.04 | 6.91 | 7.78 | 8.19 |  |
| Total | 10,485 |  |  |  |  |  | 9,994 |  |  |  |  |  |  |
| To estimate centiles below 33 weeks’ gestation, the Newborn Cross-Sectional Study sample (3) was supplemented by including neonates from the same population who, despite being born to mothers with some risk factors for fetal growth restriction (except smoking and severe obesity), did not have congenital malformations or antenatal ultrasound evidence of fetal growth restriction (6). Centiles below 28 weeks’ gestation should be interpreted with caution given the small sample size. | | | | | | | | | | | | |  |

| **Table S5: Z-scores for weight-length ratio according to gestational age of newborns enrolled in the Newborn Cross-Sectional Study of the INTERGROWTH-21^st^ Project (3, 6)** | | | | | | | | | | | | | | | | | |
| --- | --- | --- | --- | --- | --- | --- | --- | --- | --- | --- | --- | --- | --- | --- | --- | --- | --- |
| Gestational age at birth  (weeks) | Z-scores for weight-for-length ratio (Kg/m) | | | | | | | | | | | | | | | | |
|  | Number of observations | Boys | | | | | | | Number of observations | Girls | | | | | | | |
|  |  | -3SD | -2SD | -1SD | 0SD | 1SD | 2SD | 3SD |  | -3SD | -2SD | -1SD | 0SD | 1SD | 2SD | 3SD |  |
| 24 weeks | 6 | 0.51 | 1.11 | 1.71 | 2.31 | 2.90 | 3.50 | 4.10 | 8 | 0.38 | 0.97 | 1.57 | 2.17 | 2.77 | 3.36 | 3.96 |  |
| 25 weeks | 13 | 0.62 | 1.22 | 1.82 | 2.41 | 3.01 | 3.61 | 4.21 | 10 | 0.48 | 1.08 | 1.51 | 2.28 | 2.87 | 3.47 | 4.07 |  |
| 26 weeks | 15 | 0.76 | 1.36 | 1.96 | 2.55 | 3.15 | 3.75 | 4.35 | 9 | 0.62 | 1.22 | 1.65 | 2.41 | 3.01 | 3.61 | 4.21 |  |
| 27 weeks | 14 | 0.93 | 1.53 | 2.13 | 2.72 | 3.32 | 3.92 | 4.52 | 12 | 0.79 | 1.39 | 1.82 | 2.59 | 3.18 | 3.78 | 4.38 |  |
| 28 weeks | 22 | 1.14 | 1.73 | 2.33 | 2.93 | 3.53 | 4.12 | 4.72 | 19 | 1.00 | 1.60 | 2.03 | 2.79 | 3.39 | 3.99 | 4.58 |  |
| 29 weeks | 22 | 1.38 | 1.98 | 2.57 | 3.17 | 3.77 | 4.37 | 4.96 | 24 | 1.24 | 1.84 | 2.27 | 3.03 | 3.63 | 4.23 | 4.83 |  |
| 30 weeks | 28 | 1.66 | 2.26 | 2.86 | 3.45 | 4.05 | 4.65 | 5.25 | 29 | 1.52 | 2.12 | 2.55 | 3.31 | 3.91 | 4.51 | 5.11 |  |
| 31 weeks | 41 | 1.98 | 2.58 | 3.18 | 3.77 | 4.37 | 4.97 | 5.57 | 35 | 1.84 | 2.44 | 2.87 | 3.64 | 4.23 | 4.83 | 5.43 |  |
| 32 weeks | 53 | 2.35 | 2.94 | 3.54 | 4.14 | 4.74 | 5.33 | 5.93 | 48 | 2.21 | 2.81 | 3.24 | 4.00 | 4.60 | 5.20 | 5.79 |  |
| 33 weeks | 33 | 2.25 | 3.00 | 3.74 | 4.48 | 5.23 | 5.97 | 6.71 | 17 | 2.45 | 3.13 | 3.81 | 4.49 | 5.18 | 5.86 | 6.54 |  |
| 34 weeks | 48 | 2.76 | 3.49 | 4.23 | 4.96 | 5.70 | 6.43 | 7.17 | 65 | 2.85 | 3.53 | 4.21 | 4.89 | 5.57 | 6.25 | 6.94 |  |
| 35 weeks | 128 | 3.20 | 3.92 | 4.65 | 5.38 | 6.11 | 6.83 | 7.56 | 111 | 3.23 | 3.91 | 4.59 | 5.27 | 5.95 | 6.63 | 7.31 |  |
| 36 weeks | 320 | 3.59 | 4.31 | 5.03 | 5.74 | 6.46 | 7.18 | 7.90 | 292 | 3.57 | 4.25 | 4.93 | 5.62 | 6.30 | 6.98 | 7.66 |  |
| 37 weeks | 849 | 3.93 | 4.64 | 5.35 | 6.06 | 6.77 | 7.49 | 8.20 | 799 | 3.89 | 4.57 | 5.25 | 5.93 | 6.61 | 7.29 | 7.97 |  |
| 38 weeks | 2,031 | 4.23 | 4.93 | 5.64 | 6.34 | 7.04 | 7.75 | 8.45 | 1,786 | 4.17 | 4.85 | 5.53 | 6.21 | 6.89 | 7.57 | 8.25 |  |
| 39 weeks | 2,983 | 4.50 | 5.19 | 5.89 | 6.58 | 7.28 | 7.97 | 8.66 | 2,846 | 4.41 | 5.09 | 5.77 | 6.45 | 7.13 | 7.81 | 8.50 |  |
| 40 weeks | 2,531 | 4.73 | 5.41 | 6.10 | 6.79 | 7.47 | 8.16 | 8.85 | 2,486 | 4.61 | 5.29 | 5.97 | 6.65 | 7.33 | 8.01 | 8.69 |  |
| 41 weeks | 1,146 | 4.93 | 5.61 | 6.29 | 6.96 | 7.64 | 8.32 | 9.00 | 1,180 | 4.76 | 5.44 | 6.12 | 6.80 | 7.49 | 8.17 | 8.85 |  |
| 42 weeks | 202 | 5.10 | 5.77 | 6.44 | 7.11 | 7.78 | 8.46 | 9.13 | 218 | 4.86 | 5.55 | 6.23 | 6.91 | 7.59 | 8.27 | 8.95 |  |
| Total | 10,485 |  |  |  |  |  |  |  | 9,994 | 2.45 | 3.13 | 3.81 | 4.49 | 5.18 | 5.86 | 6.54 |  |
| To estimate z-scores below 33 weeks’ gestation, the Newborn Cross-Sectional Study sample (3) was supplemented by including neonates from the same population who, despite being born to mothers with some risk factors for fetal growth restriction (except smoking and severe obesity), did not have congenital malformations or antenatal ultrasound evidence of fetal growth restriction (6). Centiles below 28 weeks’ gestation should be interpreted with caution given the small sample size. | | | | | | | | | | | | | | | | | |
